# Associations of Hyperactivity and Inattention Scores with Theta and Beta Oscillatory Dynamics of EEG in Stop-Signal Task in Healthy Children 7–10 Years Old

**DOI:** 10.3390/biology10100946

**Published:** 2021-09-22

**Authors:** Andrey V. Bocharov, Alexander N. Savostyanov, Helena R. Slobodskaya, Sergey S. Tamozhnikov, Evgeny A. Levin, Alexander E. Saprigyn, Ekaterina A. Proshina, Tatiana N. Astakhova, Ekaterina A. Merkulova, Gennady G. Knyazev

**Affiliations:** 1 Laboratory of Psychophysiology of Individual Differences, Scientific Research Institute of Neurosciences and Medicine, 630117 Novosibirsk, Russia; a.n.savostyanov@physiol.ru (A.N.S.); stam@physiol.ru (S.S.T.); saprigyn@physiol.ru (A.E.S.); proshinaea@physiol.ru (E.A.P.); merkulovaea@physiol.ru (E.A.M.); knyazev@physiol.ru (G.G.K.); 2 Humanitarian Institute, Novosibirsk State University, 630090 Novosibirsk, Russia; tastahova95@yandex.ru; 3 Laboratory of Psychological Genetics, Institute of Cytology and Genetics of SB RAS, 630090 Novosibirsk, Russia; 4 Department of Child Development and Individual Differences, Scientific Research Institute of Neurosciences and Medicine, 630117 Novosibirsk, Russia; hslob@physiol.ru; 5 Institute of Medicine and Psychology, Novosibirsk State University, 630090 Novosibirsk, Russia; 6 Research Department of Angioneurology and Neurosurgery, Institute of Oncology and Neurosurgery, Meshalkin National Medical Research Center, 630055 Novosibirsk, Russia; e_levin@meshalkin.ru

**Keywords:** Stop-Signal task, theta oscillations, beta oscillations, ADHD, hyperactivity, inattention, children

## Abstract

**Simple Summary:**

Most studies on ADHD have been focused on the comparisons between healthy subjects and clinical patients. The dimensional approaches propose that the main pathological behavioral domains are distributed in the normal population and not only in individual categories of people (as assumed in traditional schemes of comparisons between patients and controls). In the current study, we used a similar approach to identify potential markers of ADHD by studying the EEG dynamics of healthy children with a natural variability in hyperactivity and inattention scores during performance of the Stop-Signal task. We found that hyperactivity/inattention scores were positively associated with RT variability. Hyperactivity/inattention scores were negatively associated with an increase in beta spectral power in the first 200 ms and positively associated with an increase in theta rhythm at about 300 ms after presentation of the Go stimulus. It has been hypothesized that such results imply insufficient vigilance in the early stages of perception and subsequent compensatory enhancing of attention to the stimulus in children with higher hyperactivity and inattention scores.

**Abstract:**

In the current study, we aimed to investigate the associations between the natural variability in hyperactivity and inattention scores, as well as their combination with EEG oscillatory responses in the Stop-Signal task in a sample of healthy children. During performance, the Stop-Signal task EEGs were recorded in 94 Caucasian children (40 girls) from 7 to 10 years. Hyperactivity/inattention and inattention scores positively correlated with RT variability. Hyperactivity/inattention and inattention scores negatively correlated with an increase in beta spectral power in the first 200 ms after presentation of the Go stimulus. Such results are in line with the lack of arousal model in ADHD children and can be associated with less sensory arousal in the early stages of perception in children with symptoms of inattention. The subsequent greater increase in theta rhythm at about 300 ms after presentation of the Go stimulus in children with higher inattention scores may be associated with increased attention processes and compensation for insufficient vigilance in the early stages of perception.

## 1. Introduction

Attention-deficit/hyperactivity disorder (ADHD) is characterized as excessive physical activity that is often combined with impaired attention. Symptoms of ADHD begin in childhood and may persist into adulthood; they are more commonly diagnosed in boys than in girls. There are three subtypes of attention-deficit/hyperactivity disorder: primarily hyperactive and impulsive, primarily inattentive, and combined [1].

Most of the research on the electrocortical activity of ADHD in children has been conducted using the ERP (event-related potential) method, which is an important tool for evaluating information processing in both normal and pathological conditions. ERP studies have revealed differences between ADHD subjects and control groups in neural processing associated with automatic early attention processes. Reduced amplitude of the P2 and N2 peaks was found in subjects with ADHD symptoms, which may be from the hypoactivation of the early orienting processes and the lack of early attention required for processing stimuli [2,3,4,5,6]. Moreover, it has been shown that differences in the early peaks of the ERP (especially in its P2 and N2 components) at the central electrodes in the time interval around 150–300 ms after presenting visual as well as auditory stimuli can distinguish ADHD children from control groups [2,7]. Differences in the later peaks of ERPs were also found [2]. According to the recent meta-analysis of ERP studies, it has been suggested that the P300 component, connected to conscious stimulus distinction, can be considered to be the most sensitive ADHD biomarker [8].

Oscillatory patterns of EEGs under resting conditions in children with ADHD have also been well studied. Earlier studies revealed that, compared to control groups, ADHD children showed a consistent pattern of an increase in theta oscillations in the frontal and central regions and a decrease in alpha and beta oscillations in the posterior and temporal regions [9,10]. A recent neurofeedback study revealed a decrease in resting state alpha power (8–12 Hz), suggesting cortical hyperactivation in adult ADHD patients, whereas an increase in alpha power during the Go/No-Go task in ADHD patients after neurofeedback training could indicate an improvement in inhibitory control [11].

To some extent, the oscillatory patterns of the background EEGs in children with symptoms of inattention and hyperactivity may reflect deviating trajectory and delay in brain development. According to the concept of normal brain development, maturation of the cortex begins in the occipital–parietal regions of the brain and continues in the temporal and frontal regions [12]. In terms of the EEG frequency spectrum, the human brain develops from a dominance of slow-wave activity (delta and theta rhythms) to an increase in the powers of the alpha and beta rhythms [13]. According to Fernandez et al. (2009), the excess of slow-wave activity in children with ADHD may be associated with delayed maturation of the frontal cortex [14]. At the same time, the lack of high-frequency oscillations in the posterior areas of the cortex in children with ADHD may indicate its insufficient development [15,16].

It has been reported that the ratio of the theta power/beta power (theta/beta ratio) of the EEG at rest could be considered to be a sensitive ADHD biomarker for differentiation of subjects with ADHD from the control group (for review see Barry et al., 2003 and McVoy et al., 2019) [9,17]. However, Coolidge, Starkey, and Cahill (2007) reported that the theta/beta ratio is not very specific for ADHD, and this EEG index showed low accuracy for differentiation of subjects with ADHD from other non-ADHD psychiatric disorder patients [18].

To summarize, the resting state EEG oscillatory pattern and ERP characteristics have been well studied for ADHD, but there are few data on EEG oscillatory responses in children with ADHD symptoms when they perform tasks requiring elevated attention and involving behavioral control mechanisms. Overall, studies of changes in the EEG oscillatory dynamics accompanying behavioral and cognitive task performance in ADHD are still underrepresented in the literature, and there is no consistency in these studies. Some studies found changes in different frequency bands (especially theta) between subjects with ADHD and control groups, others revealed no differences [19,20,21,22]. Such inconsistency in the data may be caused by the different levels of specificity regarding the ADHD condition in various experimental tasks.

One of the most popular tests in ADHD research, whose several indexes are reliably associated with the disorder, is the Stop-Signal task [23,24,25]. This task has shown its efficiency in assessing the processes of attention and motor control. The experimental task includes a mix of Go and Stop trials. Go trials require quick reactions to presented stimuli, just as in a classic choice reaction time task. In Stop trials, which have lower frequency than Go trials, a Go stimulus is followed by a Stop signal at which time participants are asked to inhibit their impending action [26].

Most studies on ADHD have been focused on the comparisons between healthy subjects and clinical patients. At the same time, the identification of the neurofunctional markers of the disorders is often hampered by serious confounding factors inherent in clinical samples of patients, such as the effects of medicines used, complex interactions between comorbidities, and disorder-specific brain structural changes [27]. These problems have led investigators to argue that using only traditional strategies for studying disorders based on comparing healthy subjects with patients may not reveal valid biomarkers. The presence of such problems has initiated the development of such dimensional disorder concepts as the Research Domain Criteria framework [28]. The main idea behind the dimensional disorders concept is that the main pathological behavioral domains are distributed in the normal population and not only in distinct categories of people (as assumed in traditional schemes of comparisons between patients and controls). In the current study, we use a similar approach to identify potential markers of ADHD by studying healthy children with varying degrees of predisposition to impaired attention and behavioral self-control.

The aim of the current study was to explore the associations between the natural variability of hyperactivity and inattention scores and the EEG oscillatory responses in the Stop-Signal task in a sample of healthy children.

The oscillations of the theta and beta ranges of the resting state EEG have been shown to be the most sensitive indicators of ADHD symptoms (for review see [17]), and both theta and beta rhythms are associated with attention and movement execution processes [29,30,31]. We hypothesized that, for the Stop-Signal task, event-related oscillatory responses within the same ranges could be related to hyperactivity and inattention scores in healthy children.

## 2. Materials and Methods

### 2.1. Participants

A total of 94 healthy Caucasian children (40 girls and 54 boys, 7 to 10 years old, mean age 8.6 ± 1.1) took part in the study. The sample was recruited from Novosibirsk, the third largest city in Russia. Typically developing children were recruited through schools in the period from September to May by the school psychologist. Children were right-handed and had normal or corrected-to-normal vision. Hyperactivity/inattention scores were measured using the parents’ reports of the Strengths and Difficulties Questionnaire (SDQ) [32]. The SDQ consists of 5 scales and 25 items (each scale includes 5 items). The Russian version of the SDQ shows good reliability and validity [33]. In the current study, the hyperactivity/inattention scale had an internal consistency Cronbach’s α = 0.79. Data collection was performed by the experimenter and psychologist at the Laboratory of Psychophysiology of Individual Differences of the Scientific Research Institute of Neurosciences and Medicine in the period from September to May.

### 2.2. Stop-Signal Task

The Stop-Signal paradigm is one of the most popular approaches to assessing response inhibition function using behavioral measures. In this paradigm, subjects perform a modified choice reaction time task, in which some stimuli requiring a response (Go stimuli) are followed by another stimulus (Stop-Signal) indicating that the response should be stopped [26]. In our study, the Stop-Signal task was presented to the subjects as a computer game using the Inquisit program (Millisecond Software, Seattle, WA, USA, https://www.millisecond.com accessed on 1 September 2021). In this game, images of animals (a rabbit or a tiger) were the Go stimuli, and the response keys corresponded to the food (carrot or meat) the participant “gave” to the animal, and the “Stop” sign (red rectangle containing the word “STOP”) appeared after some of the Go stimuli and indicated that the animal should not be “fed” in the current trial. The instruction presented to the participants was the following: “During the game, a rabbit or a tiger will appear on the screen. You need to choose the appropriate food—carrot for the rabbit (button K) or meat for the tiger (button D) and press the button before the animal disappears. If a “STOP” signal appears after the animal, nothing should be pressed. If you correctly feed the animal, you will receive a point, and in case of a wrong response (incorrect choice of food or if action was carried out after the “STOP” signal), the point will be deducted.” The participants were also instructed to use left and right index fingers for pressing left (D) and right (K) keys, respectively, and to respond as fast and as accurately as possible. We had already used this type of task in our earlier study with adult participants [34] and adapted it for children more recently [35].

The Go stimuli (17 cm × 17 cm) were presented in a pseudo-random order at the center of the screen for 500 ms with the interstimulus intervals varying randomly between 3500 and 5500 ms. The images of meat and carrot were constantly displayed as reminders in the lower part of the screen at the left and right sides, respectively.

The full task consisted of 160 trials. Among them, the first 30 trials were a training session when only Go stimuli were presented. The training session data were used to calculate the average baseline Go reaction time (Go RT) for each participant. The training was followed by the main session with 96 No-Stop (only Go stimulus, 74%) and 34 Stop (Go stimulus followed by the Stop-Signal, 26%) trials mixed pseudo-randomly.

In the Stop-Signal task, the probability of failing to stop correlated positively with the Stop-Signal delay (SSD), i.e., the time interval between the Go stimulus and Stop signals [36]. To capture this effect, the SSDs were made to vary in our task. They were calculated for each participant based on his/her baseline Go RT as 10, 20, 70, and 80% of it. SSDs for each Stop trial were chosen pseudo-randomly among these delays.

The scoring system was as follows. If a response to the Go stimulus in a No-Stop trial was correct, the participant received one additional point. One point was deducted after a wrong choice of food, lack of response, or response with a too long RT (more than 750 ms) in the No-Stop trial, and if any key was pressed in the Stop trial. The trials containing a wrong choice of food and the first 30 trials (the training session) were excluded from analysis.

### 2.3. Electroencephalography (EEG) Records

The EEG of each child was recorded individually by the experimenter in a quiet, normally lit laboratory room using a cap with 64 electrodes according to the International 10–10 system. A mid-forehead electrode was the ground, a Cz electrode was a reference. The signals were amplified with a multichannel biopotential amplifier actiChamp (Brain Products GmbH, Gilching, Germany) with bandpass 0.1–100 Hz and continuously digitized at 1000 Hz. The horizontal and vertical electrooculograms were registered simultaneously. All these bioelectrical signals were recorded by a personal computer using BrainVision Recorder software (Brain Products GmbH, Gilching, Germany).

### 2.4. Data Analysis

EEG data analysis and preprocessing were performed using the EEGLAB toolbox (http://www.sccn.ucsd.edu/eeglab/ accessed on 1 September 2021). EEG artifacts originating from electrooculograms, electromyograms, and electrocardiograms were removed using the independent component analysis (ICA) method implemented in the EEGLAB. The time-frequency decomposition of the pre-treated EEG was performed using Morlet wavelets. The number of wavelet cycles was linearly increased at a frequency beginning at 1.5 cycles and capping at 8 cycles at 40 Hz.

Event-related spectral perturbations (ERSPs) were calculated to assess stimulus presentation-evoked changes in EEG spectral power. The ERSPs show mean log-transformed event-locked deviations from mean baseline EEG power at each frequency [37]. In the Go condition, the baseline time interval was 750 ms long and began at 1000 ms prior to the Go stimulus onset. The 700 ms following the Go stimulus onset were the test interval.

The partial correlation analysis, controlling for age and sex when correlating the inattention and/or hyperactivity scores with mean RT and standard deviation of RT and with the number of correct responses, was executed by the SPSS software.

Spearman’s correlations between ADHD scores (inattention and/or hyperactivity) and every point on the time-frequency plot of the ERSPs in each EEG channel were calculated. The level of significance was set at *p* < 0.005 and correction for multiple comparisons was not applied. The plots, representing time-frequency areas for which the correlation coefficients were statistically significant (*p* < 0.005), were generated using the EEGLAB toolbox. The distribution of significant correlation coefficients across the EEG channels is shown on the head maps at the top of the figures.

To exclude the influence of age and sex from the revealed correlations, additional statistical analysis of partial correlations at a significance level of *p* < 0.05 controlling for age and sex was performed correlating the averaged overall electrode ERSP measures with the inattention and/or hyperactivity scores.

## 3. Results

The mean, standard deviation (SD), minimal, and maximal measures of psychometric and behavioral data are presented in Table 1.

The partial correlation analysis controlling for age and sex revealed significant correlations of hyperactivity/inattention scores (*r* = 0.23, *p* = 0.027) and separated inattention scores (*r* = 0.227, *p* = 0.03) with the SD of the RTs in the Go condition. These results could reflect more stable RTs in children with lower hyperactivity/inattention and inattention symptoms. The results of the partial correlation analysis are presented in Table 2.

Other correlations between inattention and/or hyperactivity scores and the behavioral indicators of Go and Stop conditions under the control of age and sex were not significant.

**Figure 1:**
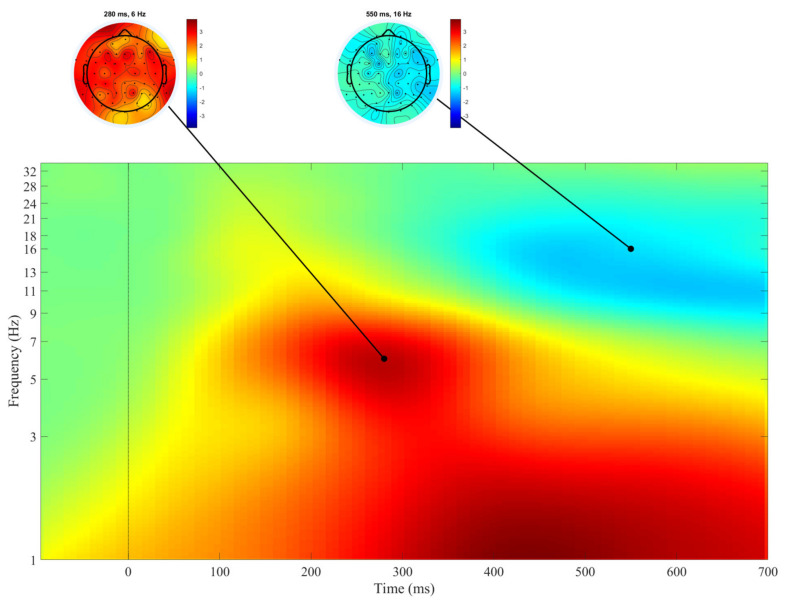
Averaged event-related spectral perturbations (ERSPs) (log test–log baseline) across all electrodes and all subjects in the Go condition. The cortical distribution of the most pronounced effects is displayed by the cortical maps at the top of the figure. An increase in spectral power is illustrated by warm colors, a decrease in spectral power by cool colors. The moment when the stimulus was presented is represented by 0. *n* = 94.

Figure 1 shows the averaged event-related spectral perturbations (ERSPs) across all cortical sites and all subjects after presentation of the Go stimulus. The most prominent features in this figure are the EEG spectral power increase in theta band, peaking at about 300 ms post-stimulus, and the spectral power decrease in the lower beta–upper alpha band starting at about 400 and lasting over 700 ms post-stimulus. The early (50–200 ms post-stimulus) spectral power increase in the lower beta–upper alpha band is also of note, but it was relatively small at the whole-group level.

The pronounced increase in theta spectral power was shown to be a typical brain reaction after detection of the stimuli, which required or evoked a subsequent reaction, not necessarily overt [29,38], and could be associated with the P300 ERP response [39]. Alpha and beta spectral power decrease (desynchronization) was observed in several studies before movement execution and even during movement observation [30,31,40].

We assumed that in the time interval beginning at 350 ms after presentation of the Go stimulus, the pronounced decrease in alpha and beta spectral power could be associated with the initiation of movement, i.e., the button press.

**Figure 2:**
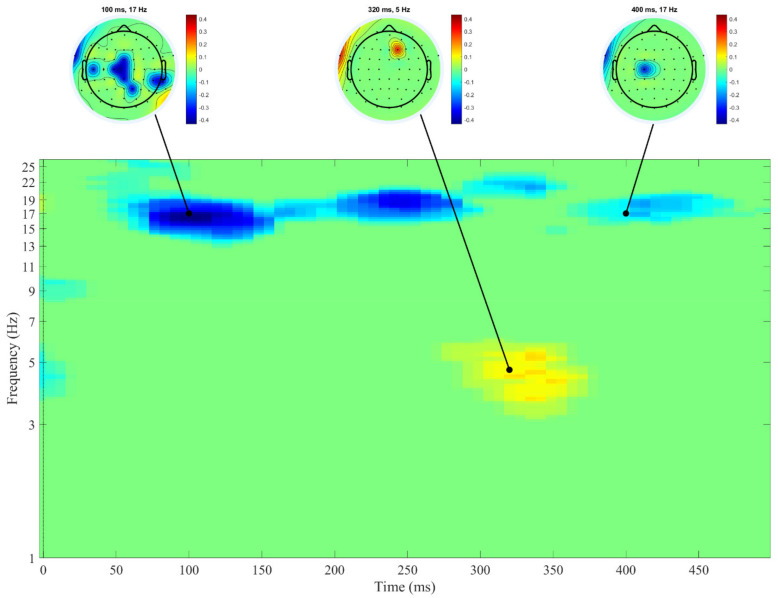
Correlations between hyperactivity/inattention scores and ERSPs after the presentation of Go stimulus. Positive correlation coefficients are shown in warm colors, negative correlation coefficients are shown in cool colors. Darker colors correspond to stronger correlations (see vertical bars for exact values). Time-frequency areas with non-significant correlation coefficients are shown in green. The moment when the stimulus was presented is represented by 0. Heads at the top of the figure display the distribution of significant correlation coefficients across the electrodes. *n* = 94.

Figure 2 shows the time-frequency distribution of the coefficients of correlations between the hyperactivity/inattention scores and the ERSP measures after presentation of the stimuli in the Go condition.

Negative correlations between hyperactivity/inattention scores and the ERSPs of the beta frequency band (from approximately 14 to 21 Hz) were observed for a time interval from 50 to 300 ms post-stimulus. Hyperactivity/inattention scores were positively correlated with an increase in theta spectral power (approximately from 3 to 6 Hz) in the time interval from 300 to 370 ms.

An additional analysis of partial correlations under the control of sex and age revealed only a negative correlation of hyperactivity/inattention scores with the ERSP measure of the beta 1 band in the time interval from 100 to 200 ms (*r* = −0.21, *p* = 0.049).

Since the hyperactivity/inattention scale includes symptoms of both hyperactivity and inattention, we divided the scale scores into those associated with hyperactivity and inattention subscales. Spearman’s and partial correlations were conducted for hyperactivity and inattention subscales and ERSP measures of the Go condition.

**Figure 3:**
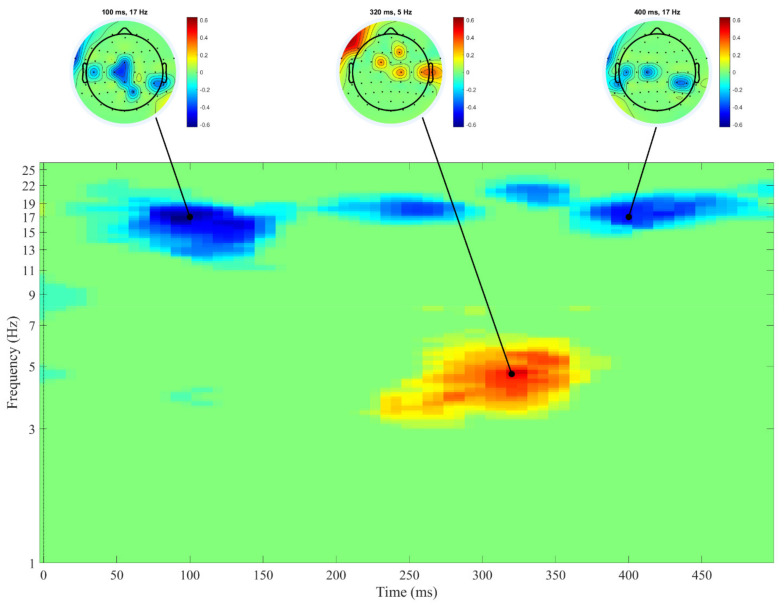
Correlations between inattention scores and ERSPs after the presentation of the Go stimulus. Positive correlation coefficients are shown in warm colors, negative correlation coefficients are shown in cool colors. Darker colors correspond to stronger correlations (see vertical bars for exact values). Time-frequency areas with non-significant correlation coefficients are shown in green. The moment when the stimulus was presented is represented by 0. Heads at the top of the figure display the distribution of significant correlation coefficients in the maps of electrodes. *n* = 94.

Figure 3 shows the time-frequency distribution of the coefficients of correlations between inattention scores and ERSP measures after the presentation of stimuli in the Go condition.

Inattention scores correlated negatively with ERSPs in the beta 1 band in the time interval of approximately 50 to 490 ms and positively—with ERSPs of the theta band in the time interval from 250 to 370 ms after the Go stimuli presentation (Figure 3).

An additional analysis of partial correlations under the control of sex and age revealed negative correlation inattention scores with ERSP measures of the beta 1 band in the time interval from 100 to 200 ms (*r* = −0.23, *p* = 0.027) and a positive correlation of inattention scores with ERSP measures of the theta band in the time interval from 250 to 350 ms (*r* = 0.24, *p* = 0.02).

The separate hyperactivity subscale was not significantly correlated under the control of sex and age with ERSP measures after presentation of the Go stimuli.

**Figure 4:**
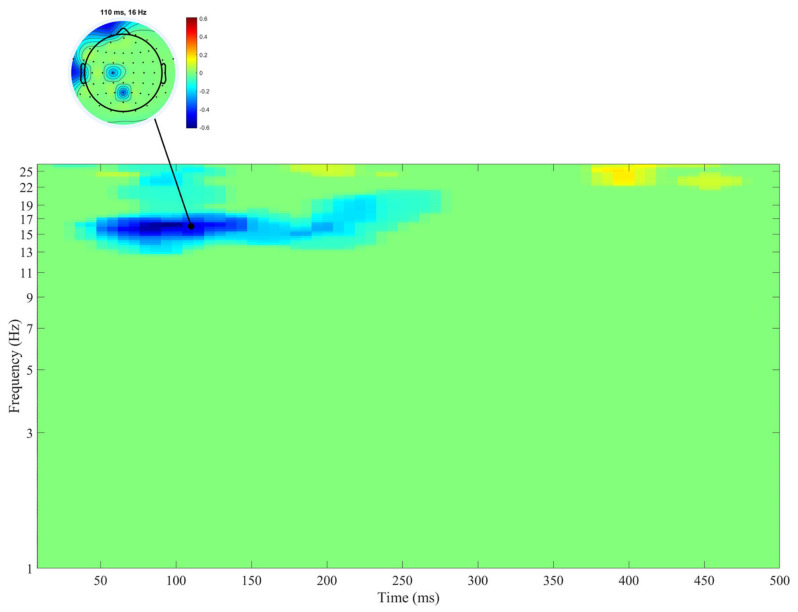
Correlations between SD RTs and ERSPs after the presentation of the Go stimulus. Negative correlation coefficients are shown in cool colors. Darker colors correspond to stronger correlations (see vertical bars for exact values). Time-frequency areas with non-significant correlation coefficients are shown in green. The moment when the stimulus was presented is represented by 0. Heads at the top of the figure display the distribution of significant correlation coefficients in the maps of electrodes. *n* = 94.

Figure 4 shows the time-frequency distribution of the coefficients of correlations between ERSP measures after the presentation of Go stimuli and SD RTs in the Go condition.

SD RTs negatively correlated with ERSPs in the beta 1 band in the time interval of approximately 50 to 200 ms after the Go stimuli presentation (Figure 4).

An additional analysis of partial correlations under the control of sex and age revealed a negative correlation of SD RTs with ERSP measures of the beta 1 band in the time interval from 100 to 200 ms (*r* = −0.21, *p* = 0.043).

An additional analysis of the partial correlations of the spectral power of the theta (4–7 Hz), alpha (8–13 Hz), and beta 1 (13–20 Hz) bands in the interstimulus interval with hyperactivity and/or inattention scores and SD RTs under the control of sex and age revealed only two negative correlations: hyperactivity/inattention scores significantly correlated with spectral power of the theta band (*r* = −0.23, *p* = 0.03) and separated inattention scores marginally significantly correlated with the spectral power of the theta band (*r* = −0.2, *p* = 0.054).

There were no significant correlations observed under the control of sex and age between inattention and/or hyperactivity scores and ERSP measures for the Stop condition.

Additional analyses were performed. A two-way ANCOVA was applied using two factors: band (two levels: beta and theta bands) and time (two levels: 0–200 and 200–400 ms) of ERSP measures of the Go condition as within-subject factors, sex as a between-subject factor, and scores of inattention and/or hyperactivity and age as covariates. We also used one-way ANOVAs to test the statistically significant differences between groups with scores below and above the median.

The interaction between hyperactivity/inattention scores, bands, and time was identified (F = 9.1, df = 1, *p* = 0.003).

The median split (median = 4) divided the sample into two groups with scores below and above the median on the hyperactivity and inattention scale. The ERSP measures of the beta range in the time interval from 0 to 200 ms after presentation of the Go stimulus were significantly higher in the group with lower scores of hyperactivity and inattention (mean = 0.57, SD = 0.5, *n* = 43) than in the group with higher scores of hyperactivity and inattention (mean = 0.34, SD = 0.5, *n* = 51) (t = 2.2, df = 92, *p* = 0.32, 95% CI lower = 0.02, 95% CI upper = 0.43). The sensitivity of beta ERSPs for the prediction of higher hyperactivity and inattention scores was 0.75.

The ERSPs of theta ranging from 200 to 400 ms after presentation of the Go stimulus were significantly lower in the group with lower scores of hyperactivity and inattention (mean = 2.1, SD = 0.9, *n* = 43) than in the group with higher scores of hyperactivity and inattention (mean = 2.3, SD = 0.9, *n* = 51) (t = −1.1, df = 92, *p* = 0.29, 95% CI lower = −0.57, 95% CI upper = 0.17). The sensitivity of theta ERSPs for the prediction of higher hyperactivity and inattention scores was 0.78.

A significant interaction between inattention, band, and time was revealed (F = 14.4, df = 1, *p* < 0.001).

The median split (median = 3) was applied to divide the sample into two groups with scores below and above the median of the separated inattention scale. The ERSPs of beta ranging from 0 to 200 ms after presentation of the Go stimulus were marginally significantly higher in the group with lower scores of inattention (mean = 0.56, SD = 0.5, *n* = 39) than in the group with higher scores of inattention (mean = 0.36, SD = 0.5, *n* = 55) (t = 1.9, df = 92, *p* = 0.59, 95% CI lower = 0.007, 95% CI upper = 0.41). The sensitivity of the beta ERSPs for the prediction of higher inattention scores was 0.84.

The ERSP measures of theta ranging from 200 to 400 ms after presentation of the Go stimulus were significantly lower in the group with lower scores of inattention (mean = 2, SD = 0.9, *n* = 39) than in the group with higher scores of inattention (mean = 2.4, SD = 0.9, *n* = 55) (t = −2.1, df = 92, *p* = 0.43, 95% CI lower = −0.75, 95% CI upper = −0.01). The sensitivity of beta ERSPs for the prediction of higher inattention scores was 0.82.

Significant interactions between the hyperactivity scores, bands, and time were not revealed. When comparing the ERSPs of the beta and theta ranges between the groups with scores below and above the median on the hyperactivity scale, no significant differences were found.

## 4. Discussion

In the current study, hyperactivity/inattention scores and inattention scores were positively associated with RT variability, which indicated less stable behavioral responses and was probably associated with reduced concentration. No associations were found between hyperactivity/inattention symptoms and separated hyperactivity and inattention with other behavioral indicators reflecting the effectiveness of motor responses and control. The results of the current study were obtained from a sample of healthy children and were similar to the results of a meta-analysis of 319 studies in which ADHD subjects did not show slower processing speed but evinced increased RT variability [24]. In this meta-analysis, Kofler and colleagues (2013) compared RT variability in the ADHD group with that in other clinical groups and revealed that higher RT variability may not be a specific ADHD sign, but a marker of a common risk factor or general psychopathology [24].

The scores of hyperactivity and inattention were not related to the error rate and reaction time; however, the natural variability of scores of hyperactivity/inattention and inattention was reflected in the pattern of oscillatory reactions. Namely, the scores of hyperactivity/inattention were correlated with oscillatory responses to stimuli presentation in the Go condition.

In an early study, Satterfield and Dawson (1971) revealed a decrease in skin conductance in children with ADHD. After administration of stimulant drugs to children with ADHD, the basal level of conductance tended to raise toward the levels of normal children. Based on these results, Satterfield and Dawson (1971) suggested that the lower basal level of arousal is inherent in ADHD [41]. Later, the idea that ADHD is related to a deficit of attention process was proposed [42]. According to the recently proposed model of regulation of vigilance, both ADHD and mania had common symptoms characterized by unstable or lowered level of vigilance due to lower level of arousal. The behavioral manifestations of ADHD and mania could be explained as an autoregulatory attempt to stabilize vigilance and increase wakefulness by creating a stimulating environment [43]. It could be suggested that such an attempt at stimulation may result in symptoms of hyperactivity.

Studies have repeatedly shown an association of beta oscillations with sensory and motor processes [30,44,45]. Moreover, according to the review of Güntekin and Basar (2014), the increase in beta rhythm was found during perception of visual stimuli that elicited higher attention and arousal [29]. According to an early hypothesis suggested by Wrobel (2000) beta oscillations can alter the visual system to higher attention level [46]. Studying both ADHD and healthy groups of children, Ogrim, Kropotov, and Hestad (2012) found that only in the healthy group was higher beta spectral power associated with a higher attention level [47].

Based on the above data, we suggest that a lowered increase in the beta spectral power (lowered beta synchronization) in the first 200 ms in children with pronounced inattention, as well as with a combination of hyperactivity and inattention, may be associated with lower sensory arousal in the early stages of perception. This hypothesis is supported by the negative correlation found between the beta spectral power in the first 200 ms after presentation of the Go stimulus and the variability in RTs under the control of sex and age (*r* = −0.21, *p* = 0.043). It has been suggested that unstable RT may be associated with a deficiency in the regulation of the internal state in ADHD, and children with ADHD symptoms must make sufficient additional efforts to adjust their hypoactivated state in accordance with the needs of the situation [19,48].

In our study, we found a positive association between theta rhythm and inattention scores, separated from hyperactivity. The revealed correlation was in accord with the results of Ogrim, Kropotov, and Hestad (2012); they found positive correlation of theta spectral power with lower attention level in both ADHD and healthy groups of children [47].

According to a review by Güntekin and Başar (2014), an increase in theta rhythm was repeatedly found during the perception of various emotionally loaded stimuli, and theta rhythm can be considered a rhythm of emotional perception and may reflect an increase of arousal [29]. It was also shown that the increase in theta spectral power, specifically frontal midline theta, was associated with increased attention, increase in cognitive load, and focused mental effort [49,50]. According to the cognitive-energetic model, primary deficits of ADHD could be related to the activation pool and effort [42]. In line with this model, additional effort could be necessary in conditions where the current state of the organism does not correspond to the state demanded for successful performance of a task [42]. It can be assumed that the increase in theta spectral power observed in our study may be associated with an increase in attention processes and compensation for insufficient vigilance in the early stages of perception. It has been shown that parietal-frontal beta [30] and frontal theta rhythms [31] are explicitly engaged not only in the movement execution processes but also in movement observation. As such, they could represent one neural correlate of social cognition, i.e., the human ability to make sense of others’ behaviors and intentions. Thus, the alterations of these rhythms could then be related to decreased social competencies. This might explain the well-known impairments of ADHD patients in social interactions [51].

The current study did not reveal significant correlations of hyperactivity and inattention scores with measures of motor response inhibition. Motor inhibition includes at least two neuropsychological components: reactive inhibitory control (the ability to inhibit a reaction forthwith after the Stop-Signal presentation) and proactive inhibitory control (the ability to change the strategy of inhibition during the SST) [52]. Van Hulst and colleagues (2018), using a modified SST, observed that ADHD children had an impaired reactive inhibitory control (greater SSRT) but an intact proactive inhibitory control [53]. They found no difference in mean RT in the Go condition between ADHD and typically developing children and suggested that between-group differences in SSRT could not be attributed to a between-group distinction in general reaction speed. However, Weigard and colleagues (2019), using a Bayesian parametric approach for unbiased evaluation of the shapes of RT and SSRT, revealed larger right skewness in both (RT and SSRT) distributions in ADHD children [25]. The authors proposed that difficulties with inhibition during SSTs in ADHD children could be related to disturbances in the early processes of attention rather than impairments in the processes of inhibition. In the current study, we could not compute a reliable estimate of the SSRT because of the limited variety of SSDs (only 10, 20, 70, and 80% of RT) and a low number of trials of the Stop-Signal condition. Further research using the Stop-Signal anticipation task [54] or the reaching arm version of the Stop-Signal task [55,56] is needed to determine the reactive and proactive inhibitory control processes in healthy children with natural variability in hyperactivity and inattention scores.

## 5. Conclusions

Hyperactivity/inattention and inattention scores negatively correlated with an increase in beta spectral power in the first 200 ms after presentation of the Go stimulus. This was probably associated with less sensory arousal in the early stages of perception in children with symptoms of inattention. These results were in line with the lack of arousal model in ADHD children. We hypothesize that the subsequent greater increase in theta rhythm at about 300 ms after the stimulus presentation in children with higher inattention scores may be associated with the increased attention processes and compensation for insufficient vigilance in the early stages of perception. According to the assumption that theta and beta rhythms reflect the human ability to make sense of others’ behaviors and intentions, the revealed alterations in these rhythms can contribute to the decrease in social competencies in children with hyperactivity/inattention symptoms.

## 6. Limitations

In the Stop-Signal task, only SSDs of 10, 20, 70, and 80% of the Go RT were used and, along with the low number of trials with the Stop-Signal presentation, this did not allow us to compute a reliable estimate of the SSRT. Therefore, we did not provide a measure of either reactive or proactive inhibitory control. Further research tackling these issues is warranted.

## Figures and Tables

**Table 1:** Psychometric and behavioral data.

	Min	Max	Mean	*SD*	Median	QR	95% CI Lower Bound	95% CI Upper Bound
Age	7	10	8.6	1.1	8.6	2	8.4	8.8
Hyperactivity/inattention SDQ scores	0	10	4.4	2.5	4	4	3.8	4.9
Separated Hyperactivity SDQ scores	0	4	1.4	1.3	1	2	1.1	1.7
Separated Inattention SDQ scores	0	6	3	1.6	3	2	2.6	3.3
Mean Go reaction time (ms)	537	702	619.6	36	622	55.5	612	627
SD of Go reaction time (ms)	34	129	80.6	15.8	80.5	19	77.4	83.9
Mean Stop-failure reaction time (ms)	481	709	593.3	55.1	594	71.5	581	604
SD of Stop-failure reaction time (ms)	2	146	77.1	26.9	76	28.5	71.5	82.7
Successful “Stop”	22%	94%	63.5%	14.4	65	20.3	60.5	66.4
Successful “Go”	34%	100%	66.7%	19.7	67.5	33.3	62.6	70.7

**Table 2:** The results of the partial correlation analysis under control of age and sex.

	Go Condition					
	SD RT		Beta		Theta	
	*r*	*p*	*r*	*p*	*r*	*p*
Hyperactivity/inattention SDQ scores	0.23	0.027	–0.21	0.049	ns	ns
Separated hyperactivity SDQ scores	ns	ns	ns	ns	ns	ns
Separated inattention SDQ scores	0.227	0.03	–0.23	0.027	0.24	0.02

ns—not significant.

## Data Availability

The data supporting reported results are available upon request from the corresponding author.
